# Identification of a juvenile-hormone signaling inhibitor via high-throughput screening of a chemical library

**DOI:** 10.1038/s41598-020-75386-x

**Published:** 2020-10-27

**Authors:** Takumi Kayukawa, Kenjiro Furuta, Keisuke Nagamine, Tetsuro Shinoda, Kiyoaki Yonesu, Takayoshi Okabe

**Affiliations:** 1 Institute of Agrobiological Sciences, National Agriculture and Food Research Organization, Ohwashi 1-2, Tsukuba, Ibaraki 305-8634 Japan; 2 Faculty of Food and Agricultural Sciences, Fukushima University, Kanayagawa 1, Fukushima, Fukushima 960-1296 Japan; 3 Drug Discovery Initiative, The University of Tokyo, Hongo 7-3-1, Bunkyo-ku, Tokyo, 113-0033 Japan

**Keywords:** Developmental biology, Physiology, Zoology

## Abstract

Insecticide resistance has recently become a serious problem in the agricultural field. Development of insecticides with new mechanisms of action is essential to overcome this limitation. Juvenile hormone (JH) is an insect-specific hormone that plays key roles in maintaining the larval stage of insects. Hence, JH signaling pathway is considered a suitable target in the development of novel insecticides; however, only a few JH signaling inhibitors (JHSIs) have been reported, and no practical JHSIs have been developed. Here, we established a high-throughput screening (HTS) system for exploration of novel JHSIs using a *Bombyx mori* cell line (BmN_JF&AR cells) and carried out a large-scale screening in this cell line using a chemical library. The four-step HTS yielded 69 compounds as candidate JHSIs. Topical application of JHSI48 to *B. mori* larvae caused precocious metamorphosis. In ex vivo culture of the epidermis, JHSI48 suppressed the expression of the Krüppel homolog 1 gene, which is directly activated by JH-liganded receptor. Moreover, JHSI48 caused a parallel rightward shift in the JH response curve, suggesting that JHSI48 possesses a competitive antagonist-like activity. Thus, large-scale HTS using chemical libraries may have applications in development of future insecticides targeting the JH signaling pathway.

## Introduction

In current pest control, integrated pest management (IPM) is a promising eco-friendly approach that combines different control methods, rather than using only chemical insecticides, to suppress pest populations to lower levels than would cause economic damage^1^. However, chemical insecticides still play a pivotal role in modern agriculture pest control and are indispensable, even in the IPM system. Recently, resistance to existing insecticides has become a major concern, and the number of usable insecticides has decreased^2–4^. Thus, the development of insecticides with novel mechanisms of action is essential for the maintenance of stable agricultural production.

Insect growth regulators (IGRs), which inhibit insect-specific development, are a class of environmentally friendly insecticides compatible with the concept of IPM^5^. Juvenile hormone (JH) is an insect-specific hormone that suppresses precocious metamorphosis during the larval stage^6^, and which has different chemical structures in different insects. Hence, JH signaling pathway may be a suitable target for developing novel IGRs with selectivity to target pests and safety to the ambient environment^5,7,8^. Indeed, JH signaling activators (JHSAs), such as JH agonists (pyriproxyfen and fenoxycarb), which suppress larval-pupal and nymph-adult metamorphosis in holometabolous and hemimetabolous insects, respectively, are often used against agricultural and sanitary insect pests^7,8^. JHSAs are efficacious in controlling small and short-lived insects, such as thrips and aphids, by impairing their metamorphosis and reducing the number of next generations. However, the application of JHSAs to the larvae of relatively large and long-lived insects, such as moths and beetles, may elongate the larval stage, thereby causing the damage to spread. In contrast, JH signaling inhibitors (JHSIs) induce precocious metamorphosis and are expected to reduce feeding-related damage to agricultural products and subsequent generations. Accordingly, JHSIs are theoretically superior to JHSAs as they have applications in the control of both long- and short-lived insect pests; however, only a few JHSIs^9^ and some inhibitors of JH biosynthesis^7^ have been reported, while none have been practically developed to date.

The molecular mechanisms of the JH signaling pathway in target cells have been elucidated in recent studies^10–14^. Briefly, JH that has entered target cells is received by a JH receptor, methoprene-tolerant protein (Met). Met proteins are a family of basic helix-loop-helix Per-ARNT-Sim (bHLH-PAS) transcription factors. Then, JH-liganded Met forms a heterodimer with steroid receptor coactivator protein (SRC), which also belongs to the family of bHLH-PAS transcription factors. The JH/Met/SRC complex activates the Krüppel homolog 1 gene (*Kr-h1*), a C_2_H_2_ zinc-finger type transcription factor, via the JH response element upstream of *Kr-h1* (*k*JHRE). Then, Kr-h1 blocks the expression of the pupal and adult specifier genes, *broad-complex* (*BR–C*) and *ecdysone-induced protein 93F* (*E93*), thereby preventing larva from precocious pupal and adult development. Based on this information, we can develop a high-throughput screening (HTS) system to conveniently evaluate the activation and inhibition activities of chemical compounds and subsequently explore novel seed compounds for JHSAs and JHSIs.

The silkworm (*Bombyx mori*) is a model lepidopteran insect with many available research tools, including genome information, transgenic methods, and cell lines^15–17^. In this study, we aimed to develop a practical JHSI by establishing a JH screening system for HTS using a *B. mori* cell line, in which the JH signaling pathway had been characterized^18–22^. Then, we carried out large-scale screening using this HTS system and succeeded in finding a novel JHSI.

## Results and discussions

### Establishment of a JH screening system

A previous study reported the identification of inhibitors of Met/SCR complex formation from plant compounds using a yeast two-hybrid system^9^. Treatment of mosquitos with these inhibitors caused defects in ovary development, whereas no effects were observed in larval development^9^. Here, we propose a screening system using an insect cell line to explore novel JHSAs and JHSIs. Because the *Kr-h1* transcript has been reported to be induced by JH in most insect cell lines^18,21,23–25^, the intrinsic factors involved in JH signaling, such as Met and SRC, are thought to be sufficiently expressed in these cell lines. In the presence of targeting insect cells, JHSA and JHSI activities were evaluated by introduction of a JH response element (JHRE)-reporter into the cells (the JHRE screening system). In this study, we established a JHRE screening system using a *B. mori* cell line as model lepidopteran insect.

**Figure 1:**
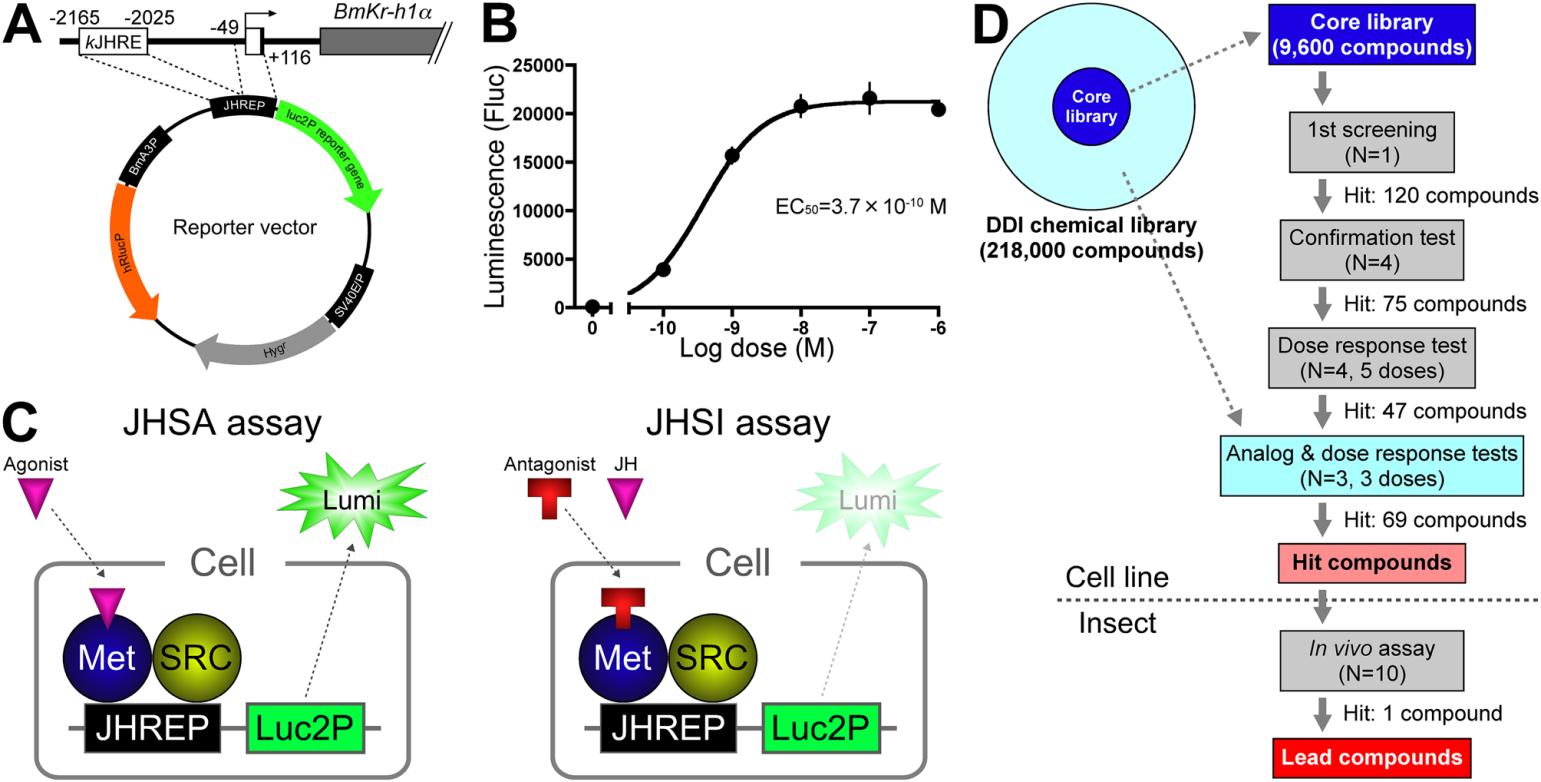
Establishment of a JH screening system and scheme of high-throughput screening (HTS). (**A**) Vector map of the reporter plasmid for the stable cell line (pGL4.14_JHREP-luc2P and BmA3P-hRlucP). *k*JHRE and the basal promoter region of *BmKr-h1* (JHREP) were inserted upstream of the firefly luciferase gene (*luc2P*), and the reference reporter gene (Renilla luciferase, *hRlucP*) was driven by the *Bombyx* cytoplasmic actin gene promoter (*BmA3P*). This plasmid was transfected into BmN cells, and a stable cell line (BmN_JF&AR) was established by selection with hygromycin. (**B**) The dose–response to JH I in BmN_JF&AR cells. BmN_JF&AR cells were treated with different concentrations of JH I, and reporter activity was examined. Data represent means ± SD (*n* = 3). (**C**) Schematic JHSA or JHSI assays using BmN_JF&AR cells. Increased reporter activity observed by adding a test compound indicated that the compound had JHSA activity (left). In the JHSI assay, JH and a test compound were simultaneously added to BmN_JF&AR cells, and the assay measured whether JH-dependent reporter activity was decreased (right). (**D**) Flow chart of HTS to identify JHSIs from the DDI chemical library using BmN_JF&AR cells. The DDI chemical library possessed 218,000 compounds in total, and the core library (9600 compounds) was composed of structurally diverse compounds for random screening. The core library was used from the first through third screenings, and analogs of the fourth screening were selected from another approximately 210,000 compounds based on the chemical structure of compounds screened in the third screening. The hit compounds selected by the four-step HTS using BmN_JF&AR cells were evaluated by topical application in *B. mori* larvae in in vivo assays.

First, we constructed a reporter vector for stable cell lines in the JHRE screening system (Fig. 1A). To reduce the background of the luc2 reporter in the JHRE reporter plasmid, as described previously (pGL4.14_− 2165 to − 2025 and − 49 to + 116)^18^, the luc2 reporter gene was swapped for a luc2P reporter gene containing the first degradation sequence (PEST)^26^. Moreover, this construct contained a hRlucP reference gene, which was continuously driven by the *BmA3* promoter^27^, as a reference reporter to evaluate the cytotoxicity of compounds (Fig. 1A).

This plasmid was transfected into *B. mori* cells (BmN cells), and the cells were selected by hygromycin for establishment of the stable line (BmN_JHRE-Fluc and A3-Rluc, BmN_JF&AR). Dose-dependent increases in reporter activities were observed in cells treated with JH I; the median effective concentration (EC_50_) was 3.7 × 10^−10^ M, whereas the reporter activity was barely detectable in the absence of JH I (Fig. 1B). This analysis clearly demonstrated that BmN_JF&AR cells were responsive to subnanomolar concentrations of natural JH I, similar to the response levels of *BmKr-h1* transcripts and transient reporter assays^18^.

Screening for JHSAs and JHSIs using this cell line is shown schematically in Fig. 1C. In the JHSA assay (left), induction of Fluc luminescence by a test compound indicates that the compound possessed JHSA activity. Meanwhile, in the JHSI assay (right), if Fluc luminescence was reduced when the cells were simultaneously treated with JH and a test compound, the compound possesses JHSI activity. False-positive results were caused by cytotoxicity of the compound and could be excluded by measurement of Rluc luminescence. In this study, we focused on exploration of JHSIs using BmN_JF&AR cells.

### HTS of JHSIs

**Figure 2:**
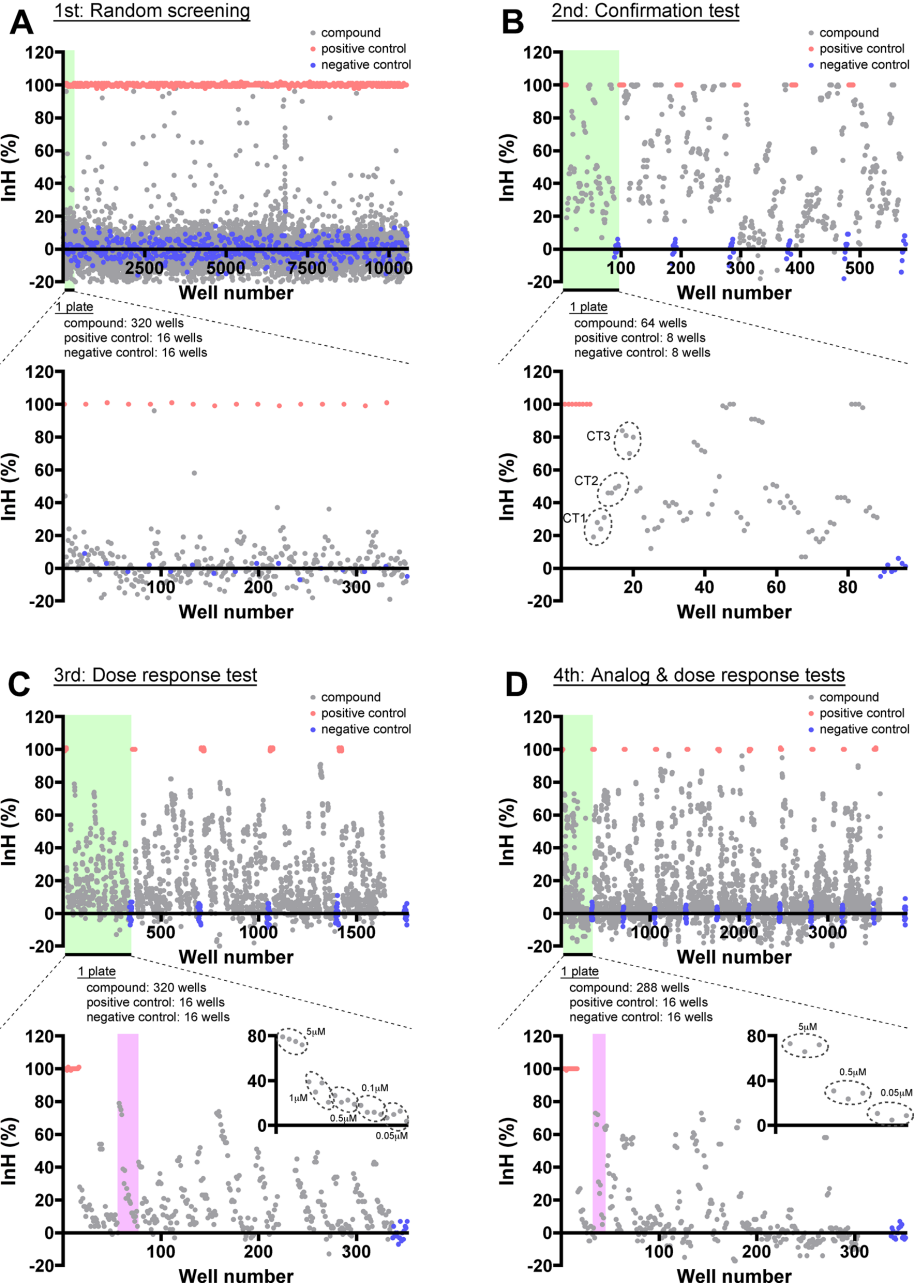
Scatter plots of HTS. JHSI activities were examined using the Dual-Luciferase reporter assay system in BmN_JF&AR cells. The JHSI activities of compounds in BmN_JF&AR cells were determined using the inhibition rate [InH (%)], where InH 0% and 100% indicated no activity and maximum JHSI activity, respectively. Gray points are wells containing 1 nM JH I and compound. The red and blue points indicate positive (only DMSO) and negative (JH I, 1 nM) controls, respectively. Plate layouts are described in Supplementary Fig. S1. (**A**) The core library (9600 compounds, 5 μM) was screened in the first screening (n = 1). The green shadow indicates a plate consisting of 320 compounds, with 16 positive control wells and 16 negative control wells, and the lower plot shows an enlarged image of a plate as an example. (**B**) The activities of compounds obtained by the first screening were confirmed in the second screening (confirmation test, n = 4). CT, compounds in confirmation test. (**C**) The dose-responses of compounds selected by the second screening were examined in the third screening (n = 4). The purple shadow focuses on a compound, and the dose–response is indicated in the inset. (**D**) Analogs of the selected compounds in the third screening were evaluated in the fourth screening (analog and dose–response tests, n = 3).

To identify JHSIs from a chemical library, we performed HTS using a four-step hit validation assay in BmN_JF&AR cells (Fig. 1D). We used 1 nM JH I in JHSI screening based on the dose–response to JH I in BmN_JF&AR cells (Fig. 1B). The plate layout used for each screening is shown in Supplementary Fig. S1. JHSI activity was calculated by the inhibition rate [InH (%)], which was evaluated according to whether a test compound inhibits the reporter activity of 1 nM JH I. Therefore, positive and negative controls were set as dimethyl sulfoxide [DMSO] alone, and 1 nM JH I in DMSO, respectively. The positive and negative controls yielded consistent results in all screenings (Fig. 2), and the performance was qualitatively assessed by Z’ factor analysis between the positive and negative controls. The average Z’ factor values of the first to fourth screenings were 0.81 ± 0.03, 0.83 ± 0.06, 0.86 ± 0.02, and 0.90 ± 0.02 (Supplementary Table S1), respectively, indicating that our screening was a highly qualitative and reproducible assay.

First, we carried out random screening with a core library consisting of structurally diverse compounds (9600 compounds, n = 1), provided by the Drug Discovery Initiative (DDI, The University of Tokyo) (Figs. 1D, 2A). The screening yielded 120 hit compounds that inhibited 1 nM JH I activity by an inhibition rate (InH) of at least 26% at a compound concentration of 5 μM (Fig. 2A). The activities of selected compounds were re-evaluated in confirmation tests during the second screening (n = 4) (Fig. 2B). Overall, the results of the first screening were highly reproducible. We excluded compounds that inhibited JH I activity at less than InH 20% or weakened cell adherence to the plate, resulting in 75 hit compounds (Fig. 2B).

Next, dose–response assays (n = 4) narrowed the hit compounds of the second screening (Fig. 2C). For example, as shown in the inset in Fig. 2C, inhibition of 1 nM JH I activity was decreased by treatment with the compounds in a concentration-dependent manner. This screening yielded 47 hit compounds with an InH of greater than 10% at 1 nM. For the fourth screening (n = 4), analogs of 47 hit compounds in the third screening were extracted from another ~ 210,000 compounds in the DDI chemical library, yielding 279 compounds. This identified approximately six analogs per hit compound in the third screening. These analogs were evaluated at three concentrations (0.05, 0.5, and 5 μM) and were selected as having InHs of greater than 15% at 0.5 μM and − 10% at 0.05 μM (Fig. 2D). Ultimately, 69 compounds were identified as candidate JHSIs that inhibited the reporter activities of 1 nM JH I in BmN_JF&AR cells (Fig. 2D).

### Characteristics of the screened compounds

**Figure 3:**
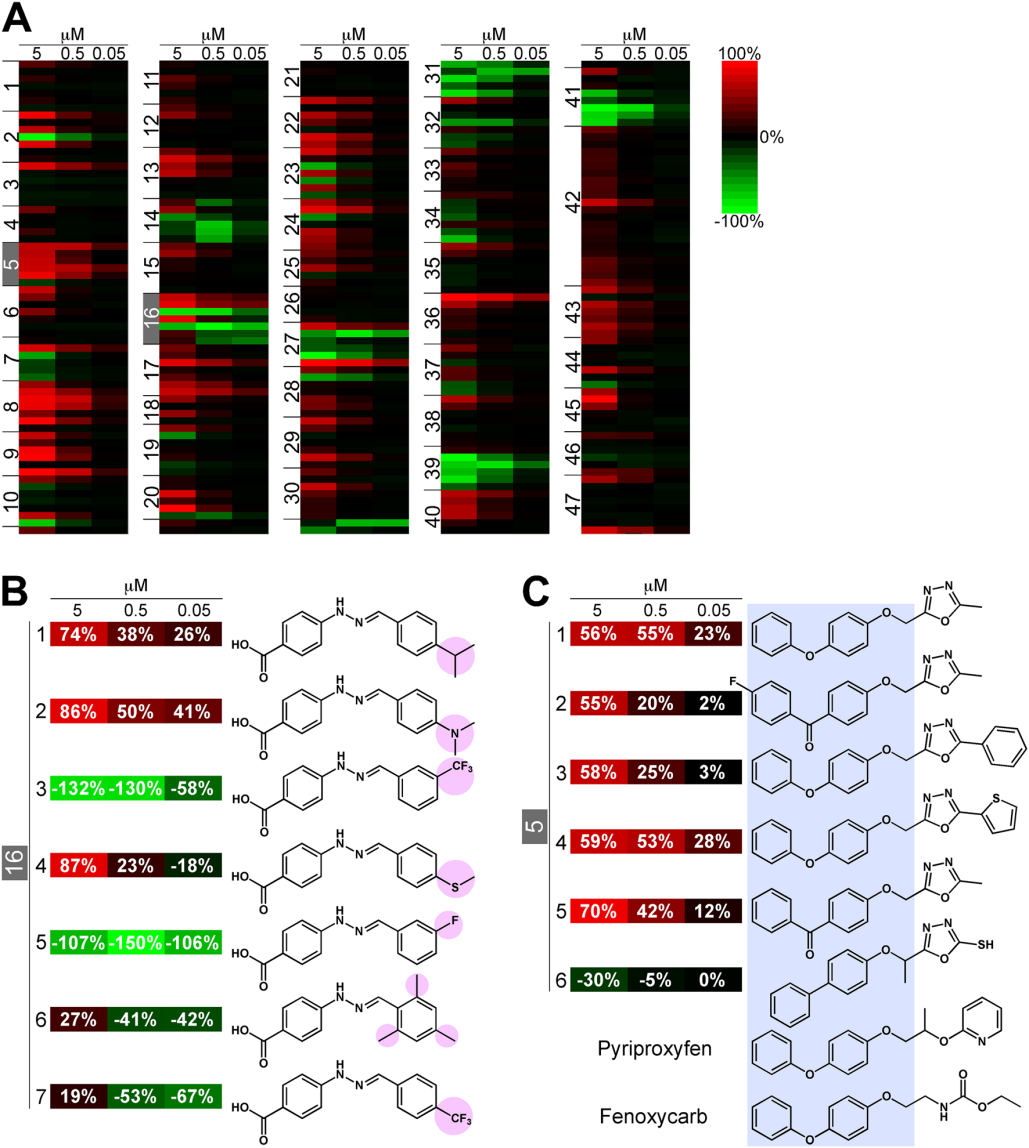
Hit compounds from HTS using BmN_JF&AR cells. (**A**) The heat map represents the results of the fourth screening. Compounds in the fourth screening were assigned numbers (analog and dose–response test [ADT] no., ADTX-Y). X numbers (left) showed the compound groups, and Y numbers show each analog in the compound group; the top line for each group (ADTX-1) show compound hits identified during the third screening, and the subsequent lines (ADTX-2, -3, -4…) show analogs of ADTX-1. Plus (red) and minus (green) values of InH (%) indicate JHSI and JHSA activities, respectively. (**B**) Differences in the functional groups converted JHSIs into JHSAs. Pink shading show differences in the functional groups. (**C**) 4-phenoxyphenoxymethyl-like compounds possessed JHSI activity. 4-phenoxyphenoxymethyl (light blue shading) was the basic structure of pyriproxyfen and fenoxycarb, two known JH agonists.

To characterize the compounds used in the fourth screening, we generated heat maps (Fig. 3A). Compounds of the fourth screening were assigned ADT numbers (ADT: analog and dose–response test), the detail for which are presented in the Fig. 3A legend. In the fourth screening, many additional analogs were observed with higher JHSI activity compared to those of the original compounds identified in the third screening, were obtained by the fourth screening, including the ADT8, ADT9, and ADT13 groups (Fig. 3A). In approximately half of the ADT groups, including ADT2, ADT7, and ADT10, the activities of the analogs were converted into JHSA activities by exchanging with slightly different functional groups (Fig. 3A). For example, ADT16-3 and ADT16-5 exhibited JHSA activities, whereas ADT16-1, ADT16-2, and ADT16-4 had JHSI activities (Fig. 3B). Interestingly, ADT5 compounds had a 4-phenoxyphenoxymethyl skeleton, similar to that of the practical JH analogs pyriproxyfen and fenoxycarb^7^, and exhibited JHSI activities (Fig. 3C).

### JHSI activity in vitro, vivo, and ex vivo

**Figure 4:**
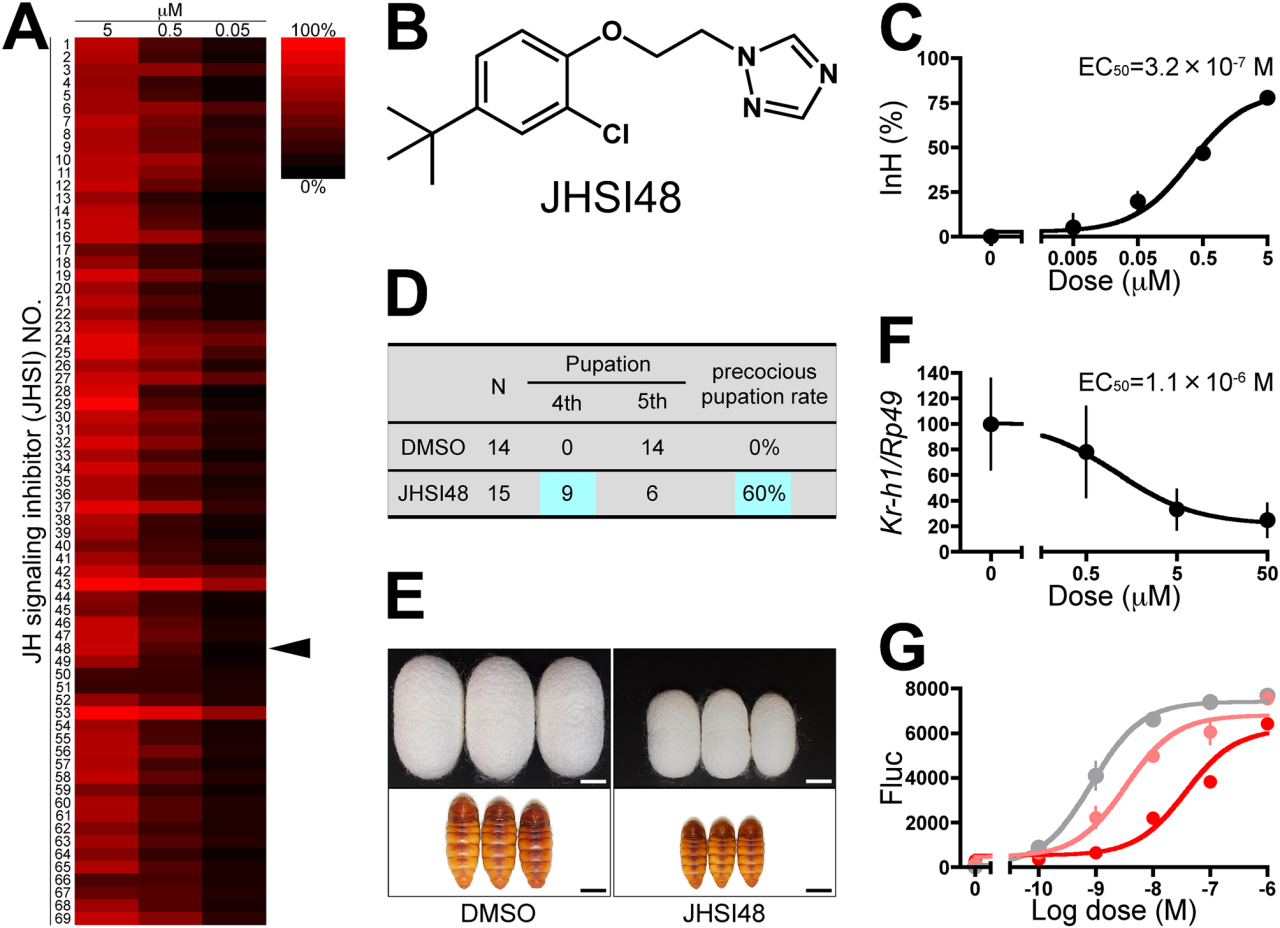
JHSI48 inhibited the JH signaling pathway in vivo. (**A**) Heat map of JHSIs extracted from the fourth screening. The JHSIs were renamed JHSI, followed by a number. The arrowhead indicates JHSI48 {*N*-[2-(4-*tert*-butyl-2-chlorophenoxy)ethyl]-1*H*-1,2,4-triazole}. (**B**) Chemical structure of JHSI48. (**C**) Dose–response of JHSI48 in BmN_JF&AR cells. BmN_JF&AR cells were treated with 1 nM JH I and different concentrations of JHSI48, and the inhibition rate (InH%) was determined 20 h after treatment. Data represent means ± SD (*n* = 3). (**D**) Effects of JHSI48 treatment on the phenotype of *B. mori*. Third instar larvae were treated with 0.5 μL DMSO (control) or JHAT48 (50 mM in DMSO) topically on 0 day, and the phenotypes were observed. (**E**) Phenotypes of cocoons and pupae treated with JHSI48. Scale bar, 1 cm. (**F**) Dose–response curve of JHSI48 ex vivo. Integuments on day 5 of the fifth instar larvae were cultured in the presence of 10 nM JH I and different concentrations of JHSI48, and *BmKr-h1* expression was monitored by qPCR. Data represent means ± SD (*n* = 10). (**G**) Pharmacological analysis of JHSI48. BmN_JF&AR cells were treated with different concentrations of JH I (0, 10^–10^, 10^–9^, 10^–8^, 10^–7^, 10^–6^ M) and JHSI48 (0, 0.5, 5 μM), after which, reporter activity was examined. Grey, 0 μM; light red, 0.5 μM; Red, 5 μM JHSI48.

Only JHSIs selected by the fourth screening were extracted from Fig. 3A and are summarized in Fig. 4A. Their chemical structures are shown in Supplementary Table S2. With regard to the cytotoxicity of the JHSIs, the highest InH (%) of Rluc luminescence was 42% for 5 μM JHSI27, while the others were all less than 20% (Supplementary Fig. S2), suggesting that no false-positive results were obtained based on our criterion (> InH 50%).

All JHSIs were topically applied to third instar larvae on day 0 of *B. mori*, and precocious metamorphosis was observed as an index of JHSI activity in vivo (n = 10). Only JHSI48 {*N*-[2-(4-*tert*-butyl-2-chlorophenoxy)ethyl]-1*H*-1,2,4-triazole} (Fig. 4B), for which the median EC_50_ was 3.2 × 10^−7^ M (Fig. 4C) in BmN_JF&AR cells, induced precocious metamorphosis at the fourth instar larvae at a rate of 60% (Fig. 4D). The cocoon and pupae became miniature sized compared to those treated with DMSO alone (control); whereas the control larvae normally molted to the fifth instar and developed into normal pupae (Fig. 4E).

Hence, we investigated the properties of JHSI48 in more detail. To exclude the possibility that precocious metamorphosis was caused by repression of JH biosynthesis in the corpora allata, we validated our findings by culture of epidermis ex vivo. A dose-dependent decrease in *BmKr-h1* transcript expression was observed in the epidermis treated with JHSI48; the median EC_50_ was 1.1 × 10^−6^ M (Fig. 4F). JH binding assays have often been performed to verify direct interactions between Met and chemicals in previous studies^28–30^. However, since commercial production of radiolabeled JH required for the assay has been discontinued, we can no longer carry this out. Thus, we carried out dose–response testing with JH I in the presence of JHSIs, a method that is often used to validate either competitive or noncompetitive antagonist in pharmacology^31,32^. According to the pharmacological principle, in the presence of the competitive antagonist, the dose–response curve is shifted to the right in a parallel manner. Alternatively, noncompetitive antagonists, such as compounds that bind to an allosteric site of the receptor or irreversibly binding to the active site of the receptor, reduce the maximal effect that can be produced by the ligand. Reduced maximal effects by JH I were observed in the presence of nine compounds (JHSI8, 23, 24, 26, 27, 28, 29, 42, and 43), while JHSI48 and the others (59 compounds) showed a parallel rightward shift in the JH response curve (Supplementary Fig. S3 and Fig. 4G). Taken together, these finding suggests that JHSI48 possesses a competitive antagonist-like activity. However, to clarify the precise action of JHSI48, further analysis is needed to demonstrate whether JHSI48 directly binds to the receptor.

## Conclusion

We established a screening system for exploration of JHSIs and found JHSI48 as a novel JHSI, which were utilized as a seed of future insecticides targeting the JH signaling pathway. Future synthetic development to improve activity could yield a practical JHSI with applications in pest management. For example, treatment of young larvae with this compound induces precocious metamorphosis and is expected to reduce feeding-related damage to agricultural products and subsequent generations. Additionally, since JH has different chemical structures in different insects, this approach may afford species specificity depending on the specific synthetic development.

## Methods

### Construction of plasmids

To reduce the background of the luc2 reporter and evaluate the cytotoxicity of compounds, the reporter plasmids pGL4.14_− 2165 to − 2025 and − 49 to + 116^18^ were reconstructed as follows. To reduce the luc2 background, a luc2P reporter gene containing PEST sequence (Promega)^26^ was swapped for the luc2 in pGL4.14_− 2165 to − 2025 and − 49 to + 116. The luc2 and luc2P fragments were digested from pGL4.14_− 2165 to − 2025 and − 49 to + 116 and a pGL4.27 plasmid (Promega), respectively, using *Hind* III and *BamH* I (Takara Bio). The digested luc2P and pGL4.14_– 2165 to – 2025 and – 49 to + 116 were ligated using a Ligation high (Toyobo) (pGL4.14_JHREP-luc2P). As a reference reporter to evaluate cytotoxicity, the *BmA3* promoter region anchored with *Kpn* I and *Bgl* II sites was amplified from the plasmid pBacA3GAL4/3 × P3DsRed^27^ using BmA3P_F and BmA3P_R primers (Supplementary Table S2). The amplified fragment was digested with *Kpn* I and *Bgl* II, and inserted into the pGL4.80 plasmid (Toyobo) (pGL4.80_BmA3P-hRlucP). The BmA3P-hRlucP region in pGL4.80_BmA3P-hRlucP was amplified using BmA3P-hRlucP_F and BmA3P-hRlucP_R primers (Supplementary Table S2) and inserted into the *Sal* I site of pGL4.14_JHREP-luc2P (pGL4.14_JHREP-luc2P and BmA3P-hRlucP).

### Establishment of a stable cell line

The BmN cell line (KATAKURA) derived from the ovaries of *B. mori* was maintained at 25 °C in IPL-41 medium (Gibco, Invitrogen) containing 10% fetal bovine serum (HyClone). BmN cells were seeded at a density of 1.5 × 10^5^ cells/well in 96-well plates 1 day before transfection. The cells were transfected with pGL4.14_JHREP-luc2P and BmA3P-hRlucP using FugeneHD (Promega). The cells were incubated for 6 h after transfection and selected with IPL-41 medium containing 100 μg/mL hygromycin (InvivoGen) for 3 months, and the stable cell line (BmN_JF&AR) was then established.

### Chemicals

JH I was purchased from SciTech. All compounds used for the HTS were supplied by the DDI of The University of Tokyo; approximately 220,000 compounds were included in the chemical library (https://www.ddi.u-tokyo.ac.jp/en/). All chemicals used in this study are commercial products, and their structures and purities were confirmed using liquid chromatography-mass spectrometry (LC–MS) at DDI.

### HTS using BmN_JF&AR cells

We used a core library from the DDI containing 9600 diverse compounds for a random screening (first screening). Analogs of compounds selected by the third screening were extracted from another ~ 210,000 compounds in the DDI chemical library and were used in the fourth screening. Compounds in DMSO were dispensed into 384- or 96-well plates using the POD Automation Platform (Labcyte). The layouts of the plates are shown in Supplementary Fig. S1. In the first, third, and fourth screenings, 10 μL medium containing BmN_JF&AR cells (final density: 5 × 10^4^ cells/well) and 10 μL of 2 nM JH I medium (final concentration: 1 nM JH I) were added to 384-well plates using a Multidrop Combi (Thermo) and incubated at 25 °C for 20 h. The treated cells were analyzed using a Dual-Luciferase Reporter 1000 Assay System (Promega) and a microplate reader (PHERAstar Plus; BMG Labtech) according to the manufacturer's instructions. In the second screening, 100 μL medium containing BmN_JF&AR cells (final density: 1.5 × 10^5^ cells/well) and 100 μL of 2 nM JH I medium (final concentration: 1 nM JH I) were added to 96-well plates, and the reporter activities were measured as described previously^18^.

The inhibition rate [InH (%)] was calculated using the following equation: InH (%) = 100 × (1 – [sample – mean_p_]/[mean_n_ – mean_p_]), where mean_p_ is the mean of Fluc or Rluc luminescence in the positive control (only DMSO), and mean_n_ is the mean of the negative control (JH I in DMSO)^33^. To evaluate the accuracy of the screenings, the Z’ factor values for each plate were calculated using the following equation; Z = 1 – (3SD_p_ + 3SD_n_)/(mean_n_ – mean_p_), where SD_n_ is the standard deviation of the negative control, and SD_p_ is the standard deviation of the positive control^34^. False-positive activity caused by the cytotoxicity of the compound was monitored by determining the InH (%) of Rluc luminescence, and compounds with greater than InH 50% were excluded from the hit compound list.

### Experimental animals and bioassays

*B. mori* (Kinsyu × Showa strain) was reared on an artificial diet (Nosan Corp) at 25 °C under a 12-h light/dark cycle. In in vivo assays, 0.5 μL hit compounds in DMSO (10 mM), which were selected by the fourth screening, were topically applied to the dorsal epidermis on day 0 of the third instar larvae, and precocious metamorphosis was observed as an index of JH antagonist activity.

### Dissection and tissue culture

Dorsal abdominal integuments on day 5 of the fifth instar larvae were dissected in phosphate-buffered saline (137 mM NaCl, 8 mM Na_2_HPO_4_, 2.7 mM KCl, and 1.5 mM KH_2_PO_4_, pH 7.4) and then cultured in Grace’s insect medium (Gibco, Invitrogen) at 25 °C, as previously described^21^. The integuments were incubated in medium containing 10 nM JH I and different concentrations of JHSI48 for 4 h.

### qPCR

Total RNA was extracted from the epidermis using an RNeasy Plus mini kit (Qiagen) and was used to synthesize cDNA with a PrimeScript RT reagent kit (Takara Bio). The primers used to quantify *BmKr-h1* (common region of two isoforms) and *BmRp49* were described previously (Supplementary Table S2)^18^. *BmRp49* was used as an internal reference. Reactions were performed in a 10-μL volume containing template cDNA derived from 1 ng total RNA, 5 μL SYBR Premix Ex Taq (Takara Bio), and each primer (0.2 μM) using a LightCycler 480 real-time thermal cycler (Roche). The PCR conditions were as follows: 95 °C for 5 min, followed by 55 cycles of 95 °C for 5 s and 60 °C for 20 s. Relative gene expression was determined by the 2^−ΔΔCt^ method^35^.

### Identification of competitive and non-competitive antagonist

To verify whether JHSIs are competitive or non-competitive antagonists, pharmacological assays^31,32^ via dose–response testing in the presence of JHSIs were carried out by the same method as described for HTS. JH I was added at concentrations of JH I 0, 10^–10^, 10^–9^, 10^–8^, 10^–7^, and 10^–6^ M, and JHSIs were tested at 0, 0.5, and 5 μM. To monitor the reduction in the maximal effect caused by non-competitive antagonist, the luminescence of only 10^–6^ M JH I was compared to that of 0.5 μM of JHSIs using Student’s *t* tests. *p* < 0.001 was regarded as non-competitive antagonist.

## Supplementary information

Supplementary Information 1.

Supplementary Table S2.

## References

[1] Kogan M (1998). Integrated pest management: historical perspectives and contemporary developments. Annu. Rev. Entomol..

[2] Oakeshott JG (2005). Comparing the organophosphorus and carbamate insecticide resistance mutations in cholin- and carboxyl-esterases. Chem. Biol. Interact..

[3] Rinkevich FD, Du Y, Dong K (2013). Diversity and convergence of sodium channel mutations involved in resistance to pyrethroids. Pestic. Biochem. Physiol..

[4] Bass C, Denholm I, Williamson MS, Nauen R (2015). The global status of insect resistance to neonicotinoid insecticides. Pestic. Biochem. Physiol..

[5] Staal GB (1975). Insect growth regulators with juvenile hormone activity. Annu. Rev. Entomol..

[6] Riddiford, L. M. (1994) Cellular and molecular actions of Juvenile hormone I. General considerations and premetamorphic actions. *Adv. Insect Phys.* **24,** 213–274. Doi: 10.1016/S0065-2806(08)60084-3.

[7] Minakuchi C, Riddiford LM (2006). Insect juvenile hormone action as a potential target of pest management. J. Pestic. Sci..

[8] Jindra M, Bittova L (2019). The juvenile hormone receptor as a target of juvenoid “insect growth regulators”. Arch. Insect Biochem. Physiol..

[9] Lee SH (2015). Identification of plant compounds that disrupt the insect juvenile hormone receptor complex. Proc. Natl. Acad. Sci. U.S.A..

[10] Jindra M, Palli SR, Riddiford LM (2013). The juvenile hormone signaling pathway in insect development. Annu. Rev. Entomol..

[11] Belles X, Santos CG (2014). The MEKRE93 (Methoprene tolerant-Krüppel homolog 1–E93) pathway in the regulation of insect metamorphosis, and the homology of the pupal stage. Insect Biochem. Mol. Biol..

[12] Jindra M, Bellés X, Shinoda T (2015). Molecular basis of juvenile hormone signaling. Curr. Opin. Insect Sci..

[13] Truman JW, Riddiford LM (2019). The evolution of insect metamorphosis: a developmental and endocrine view. Philos. Trans. R. Soc. B.

[14] Riddiford LM (2020). A life’s journey through insect metamorphosis. Annu. Rev. Entomol..

[15] Mita K (2004). The genome sequence of silkworm, Bombyx mori. DNA Res..

[16] Tamura T (2000). Germline transformation of the silkworm *Bombyx mori* L. using a *piggyBac* transposon-derived vector. Nat. Biotechnol..

[17] Lynn DE, Harrison RL (2016). Available lepidopteran insect cell lines. Methods Mol. Biol..

[18] Kayukawa T (2012). Transcriptional regulation of juvenile hormone-mediated induction of Kruppel homolog 1, a repressor of insect metamorphosis. Proc. Natl. Acad. Sci. U.S.A..

[19] Kayukawa T (2014). Hormonal regulation and developmental role of Krüppel homolog 1, a repressor of metamorphosis, in the silkworm *Bombyx mori*. Dev. Biol..

[20] Kayukawa T, Shinoda T (2015). Functional characterization of two paralogous JH receptors, methoprene-tolerant 1 and 2 in the silkworm, *Bombyx mori* (Lepidoptera: Bombycidae). Appl. Entomol. Zool..

[21] Kayukawa T (2016). Krüppel homolog 1 inhibits insect metamorphosis via direct transcriptional repression of *Broad-Complex*, a pupal specifier gene. J. Biol. Chem..

[22] Kayukawa T, Jouraku A, Ito Y, Shinoda T (2017). Molecular mechanism underlying juvenile hormone-mediated repression of precocious larval-adult metamorphosis. Proc. Natl. Acad. Sci. U.S.A..

[23] Zhang Z, Xu J, Sheng Z, Sui Y, Palli SR (2011). Steroid receptor co-activator is required for juvenile hormone signal transduction through a bHLH-PAS transcription factor, methoprene tolerant. J. Biol. Chem..

[24] Kayukawa T, Tateishi K, Shinoda T (2013). Establishment of a versatile cell line for juvenile hormone signaling analysis in *Tribolium castaneum*. Sci. Rep..

[25] Roy A, Palli SR (2018). Epigenetic modifications acetylation and deacetylation play important roles in juvenile hormone action. BMC Genom.

[26] Li X (1998). Generation of destabilized green fluorescent protein as a transcription reporter. J. Biol. Chem..

[27] Uchino K (2006). Evaluating promoter sequences for trapping an enhancer activity in the silkworm *Bombyx mori*. J. Insect Biotechnol. Sericol..

[28] Touhara K, Lerro KA, Bonning BC, Hammock BD, Prestwich GD (1993). Ligand binding by a recombinant insect juvenile hormone binding protein. Biochemistry.

[29] Miura K, Oda M, Makita S, Chinzei Y (2005). Characterization of the Drosophila Methoprene-tolerant gene product. Juvenile hormone binding and ligand-dependent gene regulation. FEBS J..

[30] Charles J-P (2011). Ligand-binding properties of a juvenile hormone receptor, Methoprene-tolerant. Proc. Natl. Acad. Sci. U.S.A..

[31] Ramsey SJ, Attkins NJ, Fish R, van der Graaf PH (2011). Quantitative pharmacological analysis of antagonist binding kinetics at CRF1 receptors in vitro and in vivo. Br. J. Pharmacol..

[32] Sum, C. S. *et al.* Pharmacological characterization of GPCR agonists, antagonists, allosteric modulators and biased ligands from HTS hits to lead optimization. *Assay guidance manual* (online). https://www.ncbi.nlm.nih.gov/books/NBK549462/ (2019). 31693331

[33] Ishii S (2017). High-throughput screening of small molecule inhibitors of the *Streptococcus* quorum-sensing signal pathway. Sci. Rep..

[34] Zhang JH, Chung TD, Oldenburg KR (1999). A simple statistical parameter for use in evaluation and validation of high throughput screening assays. J. Biomol. Screen..

[35] Livak KJ, Schmittgen TD (2001). Analysis of relative gene expression data using real-time quantitative PCR and the 2^(-ΔΔC(T))^ method. Methods.

