# Supplementary material for: Identification of a juvenile-hormone signaling inhibitor via high-throughput screening of a chemical library
Source: Sci Rep. 2020 Oct 27;10:18413. doi: 10.1038/s41598-020-75386-x (PMC7591571; doi:10.1038/s41598-020-75386-x)
Supplement: Supplementary file 1 — Supplementary Information 1. [file 41598_2020_75386_MOESM1_ESM.pdf]

Supplementary information

Identification of a juvenile-hormone signaling inhibitor via high-throughput screening of a chemical library

Takumi Kayukawa[1*], Kenjiro Furuta[1], Keisuke Nagamine[1], Tetsuro Shinoda[1], Kiyoaki Yonesu[2], Takayoshi Okabe[2*]

Supplementary Figures: Fig. S1-3.

Supplementary Tables: Table S1, 3.

*Table S2 is showed in the Excel file.

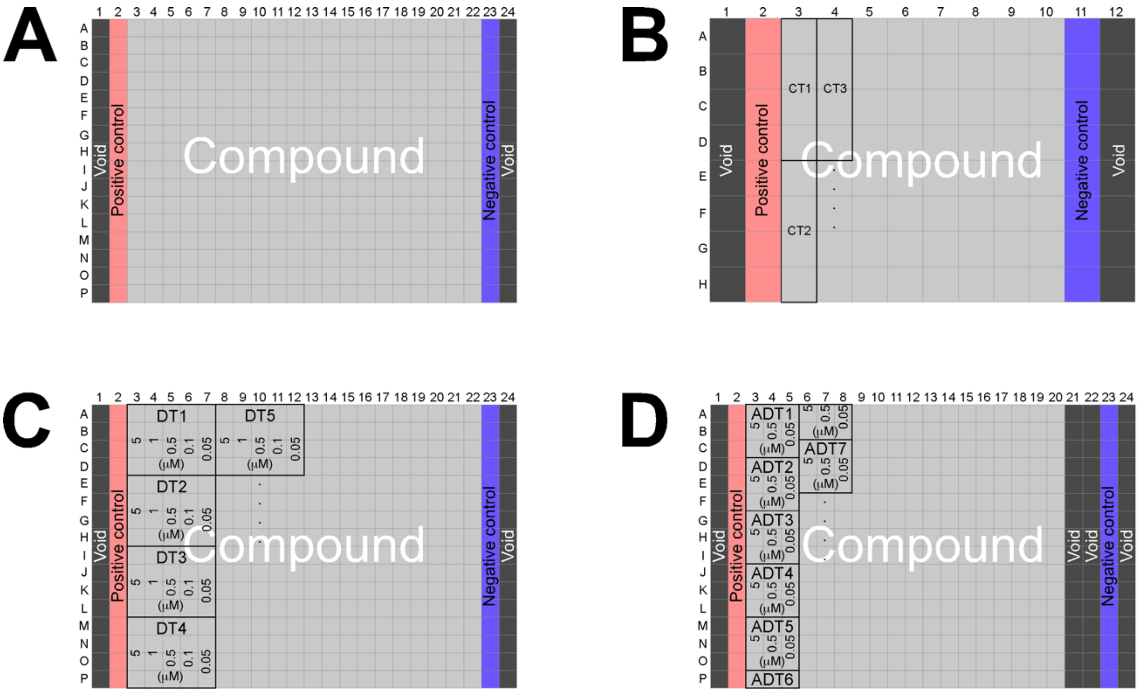

Figure S1. Layouts of plates in HTS using BmN_JF&AR cells. Gray indicates wells containing 1 nM JH I and compound. Red and blue wells indicate positive (only DMSO) and negative (JH I, 1 nM) controls, respectively, and black indicates unused wells. (A) First screening (random screening), (B) second screening (confirmation test), (C) third screening (dose-response test), and (D) fourth screening (analog and dose-response tests). CT, compound in confirmation test; DT, compound in dose-response test; ADT, compound in analog and dose-response tests.

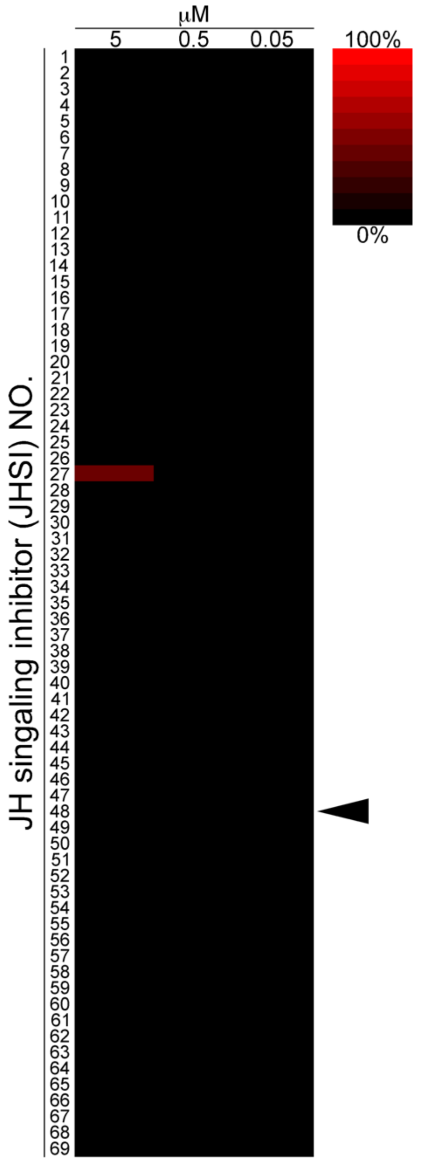

Figure S2. Cytotoxicity evaluation of JHSIs. The heat map represents the cytotoxicity evaluated by measurement of the InH (%) of Rluc luminescence. Redder suggests higher cytotoxic activities. JHSI numbers are the same in Fig. 4A.

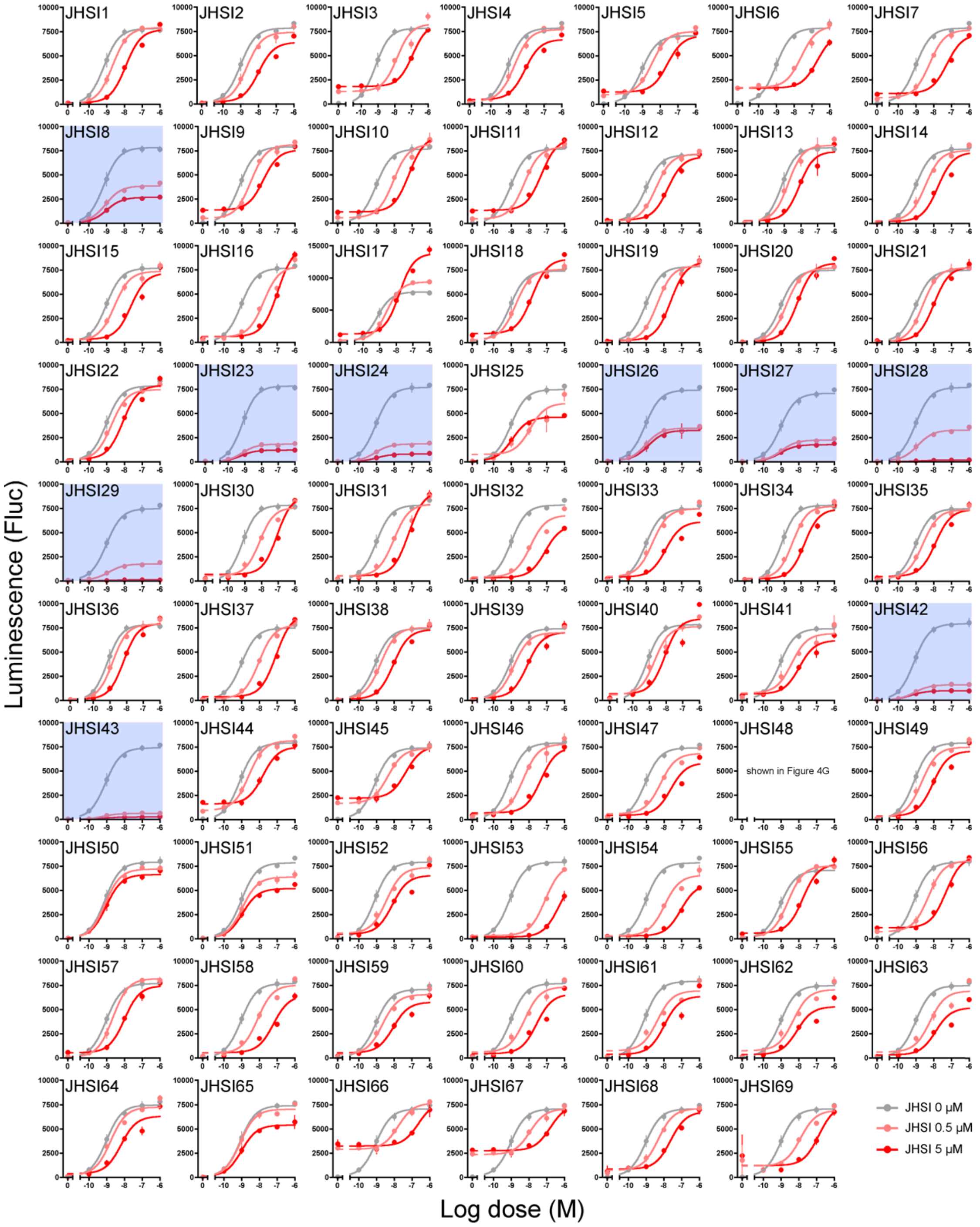

Figure S3. Pharmacological analysis of JHSIs. BmN_JF&AR cells were treated with different concentrations of JH I (0, 10^{-10}, 10^{-9}, 10^{-8}, 10^{-7}, 10^{-6} M) and JHSIs (0, 0.5, 5 μM), after which, reporter activity was examined. Grey, 0μM; light red, 0.5μM; Red, 5μM JHSIs. Blue highlighting indicates non-competitive antagonist-like activity, while all others with dose-shifts are competitive antagonists.

Table S1 Z’ factor values of HTS using BmN_JF&AR cells.

Screening name	Number of plates	Z’ factor*
First screening (random screening)	30	0.81 ± 0.03
Second screening (confirmation test)	6	0.83 ± 0.06
Third screening (dose-response test)	5	0.86 ± 0.02
Fourth screening (analog & dose-response test)	11	0.90 ± 0.02

*Data represent means ± SD.

Table S3 List of oligonucleotides used for construction and qPCR.

Name	Figure	PCR template	Nucleotide sequence (5' to 3')
**Construction**			
BmA3P	Fig. 1A	pBacA3GAL4/3×P3DsRed	TTTGGTACCTCGAGCTCAAGCTTGATGC
			TTTAGATCTCTTGAATTAGTCTGCAAGAAAAG
BmA3P-hRlucP	Fig. 1A	pGL4.80_BmA3P-hRlucP	TTTGTCGACGGTACGGGAGGTATTGGACAG
			AAAGTCGACGCCCAATACGCAAACGGATCC
**qPCR**			
BmKr-h1*	Fig. 5F	cDNA	ACCCATACTGGCGAGCGACCAT
			CCTCTCCTTTGTGTGAATACGACGG
BmRp49*	Fig. 5F	cDNA	CAGGCGGTTCAAGGGTCAATAC
			TGCTGGGCTCTTTCCACGA

*Kayukawa et al. *PNAS* 109: 11729-11734 (2012).
